# Comparative Evaluation of Sella Turcica Morphology and Dimensions in Skeletal Class III Malocclusion and Cleft Lip and Palate Patients Versus Class I Individuals

**DOI:** 10.1002/cre2.70322

**Published:** 2026-03-01

**Authors:** Bushra Gul, Erum Amin, Umar Hussain, Faiza Malik, Rana Faiza, Sundus Saad Alqarni, Waqas Naseem

**Affiliations:** ^1^ Armed Forces Institute of Dentistry Rawalpindi Pakistan; ^2^ Orthodontics Armed Forces Institute of Dentistry/National University of Medical Sciences (NUMS) Rawalpindi Pakistan; ^3^ Orthodontics Saidu College of Dentistry Swat Pakistan; ^4^ Sharif Medical & Dental College Lahore Pakistan; ^5^ Department of Orthodontics Sharif Medical and Dental College Lahore Pakistan; ^6^ College of Dentistry King Khalid University Abha Saudi Arabia; ^7^ Department of Health Sciences University of Debrecen Debrecen Hungary

**Keywords:** cleft lip and palate, dimensions, malocclusion, morphology, sella turcica, skeletal Class III

## Abstract

**Objectives:**

To compare the morphology and dimensions of the sella turcica in skeletal Class III malocclusion and cleft lip and palate (CLP) with Class I.

**Material and Methods:**

This comparative cross‐sectional study was conducted at the Armed Forces Institute of Dentistry, Rawalpindi, Pakistan, and involved 540 cases (Class I, Class III malocclusion, and CLP) using a non‐probability consecutive sampling method. The study included patients aged 12–50 years with Class I or Class III malocclusion or cleft palate while excluding those with previous orthodontic treatment or craniofacial syndromes. Data were collected from digital lateral cephalograms and patient records. The dimensions and morphology of the sella turcica were recorded. The Kruskal–Wallis rank sum test, Dunn's test, chi‐square exact test, and linear regression were applied to analyze the relationship between sella turcica dimensions and malocclusion types.

**Results:**

Sella turcica morphology differed significantly (*p* < 0.001), with normal morphology most common in Class I (58.33%) and Class III (40.00%), while oblique anterior wall predominated in CLP (30.00%). Sella turcica dimensions also varied significantly (*p* < 0.001), with Class I showing the largest median length (9.0 mm), depth (9.0 mm), and diameter (11.0 mm), followed by Class III, and the smallest values in CLP. Post hoc tests confirmed that Classes I and III had significantly greater dimensions than CLP (*p* < 0.001). Regression analysis indicated that CLP had significantly smaller length (−1.48 mm), depth (−1.23 mm), and diameter (−1.75 mm) (*p* < 0.001), while Class III showed reduced length and diameter. Males had slightly greater length and diameter, whereas age showed no significant effect.

**Conclusions:**

Class III and CLP had smaller sella turcica dimensions and more irregular shapes than Class I, suggesting that monitoring their size and shape could help identify developmental issues early for better diagnosis and treatment planning.

## 1 Introduction

The etiology of malocclusion is multifactorial, involving environmental, genetic, and epigenetic factors, and results from disharmony between the maxillary and mandibular positions in the transverse, sagittal, or vertical planes (Alhammadi et al. 2018; Mortezai et al. 2023). The sella turcica (ST) is a critical anatomical structure located on the sphenoid bone, housing the pituitary gland, and plays an essential role in craniofacial development (Sinha et al. 2020). Morphological variations and dimensional changes in the ST have been associated with several craniofacial anomalies and skeletal malocclusions (Jankowski et al. 2021). Previous studies have indicated that the morphology and size of the ST may serve as early indicators of deviations in maxillofacial growth patterns (Tepedino et al. 2019; Sönmez et al. 2024).

Skeletal Class III malocclusion is characterized by either a prognathic mandible, a retrognathic maxilla, or both (Hong and Yi 2001). The maxillary deficiency seen in Class III malocclusion is particularly pronounced in individuals with cleft lip and palate (CLP), where altered craniofacial development results from congenital defects and/or subsequent surgical interventions like lip repair (Doğan et al. 2021). Given the shared embryological origin of the midface structures and the ST from neural crest cells, anomalies in ST morphology and dimensions may plausibly reflect underlying disturbances in maxillary development, particularly in patients with skeletal Class III malocclusion and CLP (Zawiślak et al. 2023).

The cranial base, including ST, completes most of its growth by the age of 6, after which minimal changes occur in its shape and size (Currie et al. 2017). Therefore, assessment of ST morphology and dimensions can aid in the early detection of maxillary developmental abnormalities. Early identification, preferably before age 6, allows prediction of maxillary growth and timely intervention, such as maxillary protraction therapy, to guide forward growth of the maxilla (Lin et al. 2021). Early treatment may reduce the severity of malocclusion and the need for surgical intervention later, leading to improved craniofacial development and long‐term outcomes (Mandall et al. 2016).

Although numerous studies have compared ST morphology across different skeletal malocclusions in various populations (Poorsoleiman et al. 2024; Grover et al. 2023; Valizadeh et al. 2015; Alkofide 2007; Brancher et al. 2024; Shrestha et al. 2018; Magat and Ozcan Sener 2018; Afzal and Fida 2019), most have focused on Class I, Class II, and Class III relationships. However, limited evidence exists on how ST morphology and dimensions in skeletal Class III individuals compare with those in patients with CLP, both of whom commonly present with maxillary deficiency. A cone beam computed tomography (CBCT)‐based study by Akay et al. (2020) compared ST dimensions and morphology between cleft and non‐cleft individuals and found no significant differences in linear dimensions, though the inter‐clinoid distance was notably reduced in cleft patients. However, their findings were based on a relatively small sample (40 cleft and 60 non‐cleft subjects), which may limit the generalizability of their results. This study has used a relatively large sample size and so can be more generalizable.

Both skeletal Class III malocclusion and CLP are characterized by maxillary deficiency but arise from different developmental mechanisms. Skeletal Class III is mainly influenced by postnatal variations in craniofacial growth (Xue et al. 2010), whereas CLP results from early embryologic disturbances affecting craniofacial development (Selvaraj et al. 2025). Comparing these conditions helps determine whether ST morphology reflects similar maxillary deficiencies through different biological pathways. Evidence also indicates that variations in ST morphology are associated with skeletal malocclusion patterns and dental anomalies, supporting its evaluation across different craniofacial conditions, including CLP (Mortezai et al. 2023).

Recent meta‐analysis using three‐dimensional imaging indicates that non‐syndromic CLP patients present with altered ST morphology, characterized by reduced depth and diameter and a higher frequency of flattened and irregular forms compared with non‐cleft controls (Selvaraj et al. 2025). Nevertheless, the available literature is limited by methodological variability, relatively small samples, and a lack of direct comparison with defined skeletal malocclusion patterns, particularly skeletal Class III. To address these limitations, the present study evaluates ST morphology and dimensions in a large cohort including Class I, skeletal Class III, and CLP subjects using standardized cephalometric criteria. This comparative approach allows a clearer assessment of the association between ST characteristics and sagittal skeletal relationships, thereby adding clinically relevant evidence to existing literature.

Therefore, the present study aims to compare the morphology and dimensions of the ST in Class III and CLP patients relative to Class I individuals.

## 2 Materials and Methods

### 2.1 Study Design, Setting, Sampling, and Ethical Approval

This comparative cross‐sectional study was conducted at the Armed Forces Institute of Dentistry, Rawalpindi, Pakistan, on 180 cases (skeletal Class I, Class III malocclusion, and CLP) using a non‐probability consecutive sampling technique. Ethical approval was obtained from the institution's ethical committee (918/Trg/23). The data were collected from digital lateral cephalograms and patient records of individuals seeking orthodontic treatment.

### 2.2 Eligibility Criteria

The inclusion criteria were the following: patients aged 12–50 years; the presence of a unilateral or bilateral cleft; or non‐cleft patients with a skeletal Class III pattern (ANB < 2° and Wits appraisal of less than −3 mm) or Class I pattern (ANB 2°–4° and Wits appraisal 0–1 mm). Patients with a history of previous orthodontic treatment, craniofacial syndromes, trauma to the head and neck region, or a history of radiotherapy, as documented in their records, were excluded from the study. Cleft cases included individuals with unilateral or bilateral cleft lip and/or palate, diagnosed through clinical inspection of photographs, study models, and radiographs available in their records.

### 2.3 Sample Size

The initial sample size of 132 (44 participants per group: Class III, Class I, and cleft cases) was calculated using OpenEpi, based on the mean Sella midpoint height (mm) of 6.7 (SD = 1.0) in the control group and 6.1 (SD = 1.0) in the cleft group, with a 95% confidence interval and 80% power from a previous study (Antonarakis et al. 2020). To further enhance the statistical power of the study, the sample size was increased to 540 participants (180 participants per group).

### 2.4 Informed Consent

Informed consent was not required from the patients, as anonymized data containing no identifying information was used. As part of routine treatment protocols, all patients or their guardians (if under 16 years of age) had previously provided consent for the use of their data in research and publications. Age and gender were recorded from their records. All procedures were conducted in accordance with the ethical standards of the institutional research committee and with the 1964 Declaration of Helsinki and its later amendments.

### 2.5 Cephalometric Tracing

All cephalometric radiographs were acquired using the same x‐ray machine (CS 8000C, Carestream, France) within the institution. The exposure parameters were set at 85 kVp, 10 mA, with an exposure time ranging from 0.5 to 1 s. To ensure consistency, two observers verified that the radiographs were of high quality and captured with the patient in the natural head position (NHP) and lips at rest. All lateral cephalograms were manually traced by two independent examiners on acetate tracing paper using a 0.5‐mm lead pencil under standardized conditions. An illuminated viewing box was used to enhance the visualization of anatomical contours and increase tracing accuracy. The ST was traced in detail by outlining the tuberculum sellae, the floor and dorsum of the sella, and the anterior and posterior clinoid processes. Sagittal skeletal relationships were assessed by identifying Point A, nasion, and Point B for the calculation of the ANB angle. All tracings were performed following a standardized protocol to minimize measurement variability between examiners.

### 2.6 Inter‐Observer and Intra‐Observer Reliability

Two examiners (B.G. and E.A.) initially traced 20 cases, and their measurements were analyzed using the paired *t*‐test and Pearson correlation coefficient, showing almost perfect inter‐observer reliability (*r* = 0.97, *p* = 0.87). Intra‐observer reliability was also assessed by repeating measurements on the same cases after 2 weeks, demonstrating perfect consistency (*r* = 0.98, *p* = 0.82).

### 2.7 Assessment of ST Dimensions

Measurements were performed on lateral cephalograms traced manually on acetate paper, with all reference lines drawn along the median plane. The length of the ST was recorded in millimeters (mm) as the distance between the tip of the dorsum sellae and the tuberculum sellae. The depth was measured in millimeters (mm) as the perpendicular distance from this line to the deepest point of the sella floor. The diameter was determined in mm by measuring from the tuberculum sellae to the most posterior point on the inner wall of the fossa (Mortezai et al. 2023).

### 2.8 Assessing the Morphology

The morphological assessment of ST was carried out in accordance with the classification system established by Axelsson et al. (2004a). In addition to the normal morphology, five distinct variations were identified: (1) oblique anterior wall, where the anterior boundary of the sella was observed to be slanted; (2) double contour of the floor, characterized by a duplicated or layered appearance of the sella floor; (3) ST bridge, formed by excessive ossification between the anterior and posterior clinoid processes; (4) irregular notching in the posterior wall of the dorsum sellae, presenting as uneven indentations; and (5) pyramidal shape of the dorsum sellae, where the dorsum was noted to have a pointed, triangular shape.

### 2.9 Data Analysis

Data analysis was performed using R software version 4.3.1. The Shapiro–Wilk test was used to assess the normality of the continuous variables. Fisher's exact test was applied to examine the association between the morphology of ST and malocclusions. The Kruskal–Wallis rank sum test was used to compare the length, depth, and diameter of ST across different malocclusions. Dunn's test with Bonferroni correction was conducted for multiple comparisons of dimensions of ST by malocclusions. Linear regression was run using dimensions of ST as dependent variables, with type of malocclusion, age, and gender as independent variables. *p* < 0.05 was the significance level.

## 3 Results

Numerical variables such as the length, depth, and diameter of the ST were not normally distributed, as assessed by the Shapiro–Wilk test (*p* < 0.05); therefore, non‐parametric tests were used.

Table 1 shows the demographic characteristics of participants across the three groups. Both gender distribution (*p* = 0.067) and mean age (*p* = 0.080) did not differ significantly across the malocclusions.

**Table 1:** Demographics pattern of the participants.

Characteristic	Class I *N* = 180	Class III *N* = 180	Cleft lip and palate *N* = 180	*p* value*
Gender				0.067
Female	96 (53.33)	78 (43.33)	98 (54.44)	
Male	84 (46.67)	102 (56.67)	82 (45.56)	
Age (years), median (IQR)	18.0 (16.0, 20.0)	18.0 (16.0, 20.0)	17.0 (16.0, 18.5)	0.080

* Pearson's chi‐squared test; Kruskal–Wallis test.

The comparison of ST morphology among the different groups showed statistically significant differences. In Class I malocclusion (*n* = 180), the most common morphology was normal (*n* = 105, 58.33%), followed by irregular notching in the posterior wall of the dorsum sellae (*n* = 36, 20.00%) and ST bridge (*n* = 24, 13.33%). In Class III malocclusion (*n* = 180), the predominant morphology was normal (*n* = 72, 40.00%), followed by oblique anterior wall (*n* = 48, 26.67%) and ST bridge (*n* = 33, 18.33%). In the CLP group (*n* = 180), the most frequent morphology was oblique anterior wall (*n* = 54, 30.00%), followed by irregular notching in the posterior wall of the dorsum sellae (*n* = 36, 20.00%) and double contour of the floor (*n* = 27, 15.00%). The differences in ST morphology among the three groups were highly statistically significant (*p* < 0.001) (Table 2).

**Table 2:** Comparison of sella turcica morphology among various malocclusions.

Characteristic	Class I *N* = 180	Class II *N* = 180	Cleft lip and palate *N* = 180	*p* value*
Double contour of floor	6 (3.33)	3 (1.67)	27 (15.00)	< 0.001
Irregular notching in the posterior wall of dorsum sellae	36 (20.00)	9 (5.00)	36 (20.00)	
Normal	105 (58.33)	72 (40.00)	36 (20.00)	
Oblique anterior wall	3 (1.67)	48 (26.67)	54 (30.00)	
Pyramidal shape of dorsum sellae	6 (3.33)	15 (8.33)	9 (5.00)	
Sella turcica bridge	24 (13.33)	33 (18.33)	18 (10.00)	

* Pearson's chi‐squared test.

Significant differences in ST dimensions were observed among the three groups (Table 3). The median length was highest in Class I (9.0 [8.0–10.0] mm), followed by Class III (8.0 [7.0–10.0] mm) and CLP (7.0 [6.0–8.0] mm) (*p* < 0.001). Post hoc analysis showed a significant difference between Class I and Class III (*p* = 0.018), while both were significantly greater than the CLP group (*p* < 0.001). Median depth was almost similar between Class I (9.0 [8.0–9.0] mm) and Class III (9.0 [8.0–10.0] mm; *p* = 0.99). However, the depth of Class I and III was significantly greater than CLP (8.0 [6.0–9.0] mm) (*p* < 0.001). Regarding diameter, the largest median value was observed in Class I (11.0 [11.0–13.0] mm), followed by Class III (11.0 [10.0–12.0] mm) and CLP (10.0 [8.5–11.5] mm) (*p* < 0.001). Post hoc analysis showed no significant difference between Class I and Class III (*p* = 0.162), but both groups had significantly greater diameters than CLP (*p* < 0.001 for both) (Table 3).

**Table 3:** Comparison and post hoc analysis of sella turcica dimensions by type malocclusion.

Dimensions of sella turcica (mm)	Class I, *N* = 180	Class III, *N* = 180	Cleft lip and palate, *N* = 180	*p* *	Class I vs. Class III *p* **	Class I vs. CLP *p* **	Class III vs. CLP *p* **
Length	9.0 (8.0, 10.0)	8.0 (7.0, 10.0)	7.0 (6.0, 8.0)	< 0.001	0.018	< 0.001	< 0.001
Depth	9.0 (8.0, 9.0)	9.0 (8.0, 10.0)	8.0 (6.0, 9.0)	< 0.001	0.99	< 0.001	< 0.001
Diameter	11.0 (11.0, 13.0)	11.0 (10.0, 12.0)	10.0 (8.5, 11.5)	< 0.001	0.162	< 0.001	< 0.001

* Kruskal–Wallis rank sum test.

** Dunn's test with Bonferroni correction.

**Figure 1:**
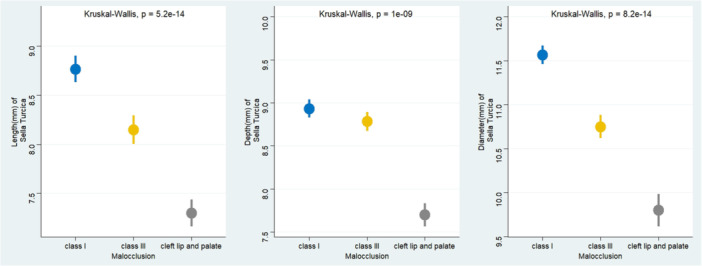
Sella turcica dimensions by type malocclusion.

ST length, depth, and diameter differed significantly among Class I, Class III, and CLP groups (*p* < 0.001). Length and diameter were highest in Class I, slightly lower in Class III, and smallest in CLP. Depth was similar between Class I and Class III but reduced in CLP. Overall, CLP patients consistently exhibited smaller sella dimensions compared with non‐cleft malocclusion groups (Figure 1).

Linear regression showed that, after adjusting for age and gender, significant associations were observed between malocclusion type and ST dimensions. Compared with Class I, individuals with CLP had significantly smaller length (−1.48 mm, *p* < 0.001), depth (−1.23 mm, *p* < 0.001), and diameter (−1.75 mm, *p* < 0.001). Those with Class III malocclusion also demonstrated significantly reduced length (−0.66 mm, *p* = 0.001) and diameter (−0.86 mm, *p* < 0.001), although the difference in depth was not statistically significant (−0.17 mm, *p* = 0.303). Gender showed a notable influence: males had significantly greater length (0.38 mm, *p* = 0.020) and diameter (+0.49 mm, *p* = 0.003) compared to females, while depth did not differ significantly (*p* = 0.112). Age was not significantly associated with any ST dimension (*p* > 0.05). The overall model fit was modest, explaining approximately 9%–13% of the variance in ST measurements (Adjusted *R* ^2^ = 0.09 for length, 0.10 for depth, and 0.13 for diameter) (Table 4).

**Table 4:** Linear regression analysis of sella turcica dimensions by malocclusion, age, and gender.

Predictor (ref)	Length of ST (mm) Estimate (SE)	*p*	Depth of ST (mm) Estimate (SE)	*p*	Diameter of ST (mm) Estimate (SE)	*p*
Intercept	9.07 (0.58)	< 0.001	8.70 (0.49)	< 0.001	10.88 (0.60)	< 0.001
Malocclusion (ref: Class I)						
Class III	−0.66 (0.20)	0.001	−0.17 (0.17)	0.303	−0.86 (0.20)	< 0.001
Cleft lip and palate	−1.48 (0.20)	< 0.001	−1.23 (0.17)	< 0.001	−1.75 (0.20)	< 0.001
Age (years)	−0.03 (0.03)	0.396	0.01 (0.03)	0.784	0.03 (0.03)	0.429
Gender (ref: Female)						
Male	0.38 (0.16)	0.020	0.22 (0.14)	0.112	0.49 (0.17)	0.003

*Note:* Length: *R* ^2^ = 0.105, adj *R* ^2^ = 0.09; depth: *R* ^2^ = 0.11, adj *R* ^2^ = 0.10; and diameter: *R* ^2^ = 0.13, adj *R* ^2^ = 0.13.

## 4 Discussion

This study found significant differences in ST dimensions (length, depth, and diameter) across three groups: Class I malocclusion had the largest dimensions, followed by Class III and CLP. Additionally, ST morphology varied, with Class I showing the most normal shapes, Class III having more bridging and oblique anterior walls, and CLP exhibiting more oblique anterior walls and double contours. These results are similar to previous studies, suggesting that genetic and developmental factors affect cranial base and ST morphology.

The ST is a key structure in orthodontics, as the sella point is used in cephalometric analyses and the ST houses the pituitary gland, which plays a vital role in craniofacial growth. It develops from neural crest cells in the front and paraxial mesoderm in the back. Abnormalities in its size or shape are associated with conditions such as spina bifida, CLP, Down syndrome, and Class III malocclusions.

In this study, we adopted the classification system proposed by Axelsson et al. to categorize ST morphology. This system identifies five distinct types of abnormalities: oblique anterior wall, ST bridging, double contour of the floor, irregular notching in the posterior dorsum, and a pyramidal shape of the dorsum. Among the Class III patients in our study, 40% had a normal sella, which aligns with the 39.13% found by Nerurkar et al. (2022). In contrast, Axelsson et al. (2004a) found that 66% of the Norwegian population had a normal sella, while Silveira et al. (2020) reported 88.4% of Brazilians had typical sella morphology. These differences in results are likely attributable to variations in ethnicity.

In this study, irregular notching in the posterior wall of the dorsum sellae was the most common abnormality in both the Class I malocclusion and CLP group. This kind of notching might happen because of small disturbances during the bone development of the ST, possibly influenced by genetic or growth‐related factors. In the Class III malocclusion group, the oblique anterior wall was seen most often. This makes sense because Class III cases usually involve jaw and facial growth differences, which could affect the front part of ST (Sathyanarayana et al. 2013). Similarly, in the CLP group, the oblique anterior wall was the most frequent finding too. Since cleft conditions often affect how the middle part of the face grows, it is not surprising that these patients also show more changes in the ST (Sundareswaran and Nipun 2015). We also noticed a higher number of double contour of the floor cases in the CLP group. This could be due to irregular bone development that happens alongside cleft formation.

This study found significant differences in the size of the ST among individuals with Class I and Class III malocclusion and CLP. Overall, people with Class I malocclusion had the largest measurements for length, depth, and diameter of ST, while those with CLP had the smallest. This pattern suggests that normal facial growth, as seen in Class I individuals, is associated with more fully developed ST (Schwab et al. 2025). In contrast, conditions like CLP, which are known to affect midfacial development, also seem to impact the cranial base, where the ST is located (Antonarakis et al. 2020). The much smaller sella dimensions in the CLP group reflect how cleft conditions can disrupt growth in many parts of the head and face, not just the mouth and nose (Alkofide 2008). While Class III individuals also had smaller ST measurements compared to Class I, the differences were less extreme than those seen with CLP. This suggests that although skeletal Class III malocclusion changes jaw positioning, it does not affect the cranial base as severely as a congenital condition like a cleft.

When we compared the groups more closely, we found that Class I and CLP groups differed significantly in both length and diameter, and Class III and CLP groups showed significant differences in length and depth. These results consistently pointed to the CLP group having smaller sella measurements in every comparison, further supporting the idea that congenital conditions can have a bigger impact on cranial development than malocclusion alone. Our findings are in line with other studies that suggest the size and shape of ST can be an early sign of craniofacial growth problems (Sathyanarayana et al. 2013; Roomaney and Chetty 2021). Clinically, noticing a smaller ST could help orthodontists and other specialists recognize underlying developmental issues early on, especially in patients with cleft conditions who often require more complex, team‐based care.

In this study, we used lateral cephalograms for assessment, while 3D imaging such as CBCT can provide more accurate measurements without magnification errors. However, CBCT was not justified in these cases, as Class I and Class III malocclusions can be diagnosed adequately using 2D imaging like cephalograms. To our knowledge, only one study has used CBCT to assess ST morphology, but that study focused on impacted teeth, for which CBCT is part of the standard diagnostic workup (Canigur Bavbek et al. 2022).

The present findings broadly align with previous meta‐analytic evidence regarding ST morphology and dimensions in individuals with CLP. Patients with CLP in this study demonstrated significantly smaller ST length, depth, and diameter compared with non‐cleft Class I and Class III individuals. For example, the observed reductions in CLP individuals (length: −1.48 mm; depth: −1.23 mm; and diameter: −1.75 mm) are comparable to the pooled effect sizes reported in the meta‐analysis for unilateral CLP (length: −1.22 mm; depth: −0.65 mm; and area: −4.75 mm^2^). The increased prevalence of morphological variations, such as oblique anterior walls and double contour of the floor in the CLP group, supports the meta‐analytic observation of a higher odds of ST bridging in cleft patients (Siddiqui et al. 2025). These results add to the pooled findings by providing a larger comparative perspective across Class I, Class III, and CLP groups within a single cohort. While previous studies primarily focused on CLP versus non‐cleft comparisons, the current study highlights graded differences across malocclusion types, showing that Class III individuals also exhibit reductions in ST length and diameter relative to Class I, although to a lesser extent than CLP patients. Furthermore, the regression analysis emphasizes the independent effects of gender on ST dimensions, an aspect not fully addressed in the prior meta‐analysis.

ST morphology and dimensions may change with age and exhibit different growth patterns between males and females. Our baseline comparisons and regression analyses indicate that the observed differences in ST morphology and dimensions across malocclusion types are robust and not confounded by age or gender.

The relatively low adjusted *R* ^2^ values (0.09–0.13) in our regression models indicate that malocclusion type accounts for only a small portion of the variation in ST dimensions. This is not unexpected, as ST morphology and size are influenced by multiple biological and developmental factors, including cranial base growth, sex, age, genetic determinants, and environmental influences (Axelsson et al. 2004b; Ominde et al. 2024). Therefore, while the associations with malocclusion are statistically significant, they should be interpreted as one of many contributing factors rather than as a strong predictor of ST dimensions.

Our findings have clinical implications, like the observed variations in ST morphology and dimensions among Class I, Class III, and CLP patients have important clinical implications. Recognition of smaller sella dimensions and atypical morphologies in CLP or Class III individuals can aid orthodontists and craniofacial specialists in early screening for potential craniofacial growth deviations. These findings may inform individualized treatment planning, such as anticipating altered cranial base development, tailoring cephalometric analyses, or adjusting the timing of orthopedic or surgical interventions. Moreover, documenting sella morphology could serve as an adjunctive diagnostic marker in longitudinal monitoring of craniofacial growth, ultimately supporting earlier and more precise clinical decision‐making.

### 4.1 Limitations

This study has a few limitations. First, we used lateral cephalograms, which only give a 2D view of the craniofacial structures. Using 3D imaging would have provided a more detailed look at the ST. Also, while a larger sample size might have strengthened the results, the homogeneity of the current sample is actually a strength. We could have also used geometric morphometric techniques to analyze the data in more detail, especially when linking sella shape to craniofacial patterns. Additionally, we did not record whether cleft patients had unilateral or bilateral CLP, nor the details of primary or secondary surgical repairs, so we cannot assess how these factors might influence ST morphology. Morphological classification is subjective, and inter‐ or intra‐observer reliability (e.g., kappa statistics) was not determined, which should be addressed in future studies. Finally, since this was a cross‐sectional study, it only gives us a snapshot at one moment in time and does not show how ST changes over time. Future research should follow participants over a longer period, include cleft type and surgical history, report reliability statistics, and use 3D imaging to better understand cranial base development.

## 5 Conclusion

People with Class III and CLP had smaller ST dimensions and more irregular shapes compared to those with Class I. Genetic and developmental factors may play a bigger role in cranial base development and, ultimately, malocclusions. Monitoring the size and shape of ST could help identify potential developmental issues early, making it easier to diagnose and plan treatment in Class III and CLP cases.

## Author Contributions

**Bushra Gul:** conceptualization (equal), data collection (equal), writing – review and editing (equal), final approval. **Erum Amin:** conceptualization (equal), data curation (equal), investigation (equal), methodology (equal), writing – review and editing (equal), final approval. **Umar Hussain:** conceptualization (equal), data analysis (equal), writing – review and editing (equal), final approval. **Faiza Malik:** conceptualization (equal), data entry (equal), writing – review and editing (equal), final approval. **Rana Faiza:** conceptualization (equal), data interpretation (equal), writing – review and editing (equal), final approval. **Sundus Saad Alqarni:** conceptualization (equal), literature review (equal), methodology (equal), writing – review and editing (equal), final approval. **Waqas Naseem:** conceptualization (equal), data analysis (equal), writing – review and editing (equal), final approval.

## Funding

The authors received no specific funding for this work.

## Ethics Statement

The study was approved by Orthodontics at the Armed Forces Institute of Dentistry, Rawalpindi, Pakistan (reference number: No. 91/Trg/23). Informed consent was not required from the patients, as anonymized data without any identifying information was used. As part of routine treatment protocols, all patients or their guardians had previously provided consent for the use of their data in research and publications. All procedures were conducted in accordance with the ethical standards of the institutional research committee and with the 1964 Declaration of Helsinki and its later amendments.

## Conflicts of Interest

The authors declare no conflicts of interest.

## Supporting information

STROBE‐checklist.

## Data Availability

Data of current study are available from the corresponding author on reasonable request. However, for privacy reasons, no individual data allowing identification of participants (e.g., videos) can be provided.
